# The Detroit Young Adult Asthma Project: Proposal for a Multicomponent Technology Intervention for African American Emerging Adults With Asthma

**DOI:** 10.2196/resprot.8872

**Published:** 2018-05-07

**Authors:** Karen MacDonell, Sylvie Naar, Wanda Gibson-Scipio, Jean-Marie Bruzzese, Bo Wang, Aaron Brody

**Affiliations:** ^1^ Department of Family Medicine and Public Health Sciences Wayne State University Detroit, MI United States; ^2^ Florida State University Tallahassee, FL United States; ^3^ College of Nursing Wayne State University Detroit, MI United States; ^4^ Columbia University New York, NY United States; ^5^ Quantitative Health Sciences Division of Biostatistics and Health Services Research University of Massachusetts Amherst, MA United States; ^6^ Department of Emergency Medicine Wayne State University Detroit, MI United States

**Keywords:** asthma, telemedicine, medication adherence, young adults, health equity

## Abstract

**Background:**

Racial and ethnic minority youth have poorer asthma status than white youth, even after controlling for socioeconomic variables. Proper use of asthma controller medications is critical in reducing asthma mortality and morbidity. The clinical consequences of poor asthma management include increased illness complications, excessive functional morbidity, and fatal asthma attacks. There are significant limitations in research on interventions to improve asthma management in racial minority populations, particularly minority adolescents and young adults, although illness management tends to deteriorate after adolescence during emerging adulthood, the unique developmental period beyond adolescence but before adulthood.

**Objective:**

The objective of the pilot study was to test the feasibility, acceptability, and signals of efficacy of an intervention targeting adherence to controller medication in African American youth (ages 18-29) with asthma. All elements of the protocol were piloted in a National Heart, Lung, and Blood Institute (NHLBI)–funded pilot study (1R34HL107664 MacDonell). Results suggested feasibility and acceptability of the protocol as well as proof of concept. We are now ready to test the intervention in a larger randomized clinical trial.

**Methods:**

The proposed study will include 192 African American emerging adults with moderate to severe persistent asthma and low controller medication adherence recruited from clinic, emergency department, and community settings. Half of the sample will be randomized to receive a multicomponent technology-based intervention targeting adherence to daily controller medication. The multicomponent technology-based intervention consists of 2 components: (1) 2 sessions of computer-delivered motivational interviewing targeting medication adherence and (2) individualized text messaging focused on medication adherence between the sessions. Text messages will be individualized based on ecological momentary assessment. The remaining participants will complete a series of computer-delivered asthma education modules matched for length, location, and method of delivery of the intervention session. Control participants will also receive text messages between intervention sessions. Message content will be the same for all control participants and contain general facts about asthma (not tailored).

**Results:**

It is hypothesized that youth randomized to multicomponent technology-based intervention will show improvements in medication adherence (primary outcome) and asthma control (secondary outcome) compared with comparison condition at all postintervention follow-ups (3, 6, 9, and 12 months). The proposed study was funded by NHLBI from September 1, 2016 through August 31, 2021.

**Conclusions:**

This project will test a brief, technology-based intervention specifically targeting adherence to asthma controller medications in an under-researched population, African American emerging adults. If successful, our multicomponent technology-based intervention aimed at improving adherence to asthma medications has the potential to improve quality of life of minority emerging adults with asthma at relatively low cost. It could eventually be integrated into clinical settings and practice to reach a large number of emerging adults with asthma.

**Trial Registration:**

ClinicalTrials.gov NCT03121157; https://clinicaltrials.gov/ct2/show/NCT03121157 (Archived by WebCite at http://www.webcitation.org/6wq4yWHPv)

## Introduction

### Background

Asthma disproportionately affects underrepresented minority populations, with African Americans having higher rates and poorer asthma outcomes than other racial and ethnic groups [1,2]. Despite growing awareness of these inequities, disparities in asthma rates and outcomes persist [3]. Age-related (or developmental) inequities also exist within minority populations, as African American adolescents and young adults have poorer asthma outcomes and higher asthma mortality rates than African American adults [2,4-8]. Overall, racial and ethnic minority youth have poorer asthma outcomes than white youth, even after controlling for socioeconomic variables [9], which may be due, in part, to differences in self-management. For example, African Americans and Latinos report low use of controller medications [9,10], often use a “crisis management style over-relying on rescue medications [10], and are nonadherent to treatment plans [10]. Proper use of medications is critical in reducing asthma mortality and morbidity [11-13]. Clinical consequences of poor asthma management include increased illness complications, excessive functional morbidity, and fatal asthma attacks [14,15].

This project focuses on African Americans in *emerging adulthood*, a unique developmental period (age 18-29 years) beyond adolescence but before adulthood [16] largely neglected in research. This represents a significant gap in knowledge, given emerging adults are at increased risk for illness consequences, even more so than adolescents. The necessary transition to adult care settings can be difficult for those with chronic conditions [17-20]. Aging out of pediatric care is often associated with increased risk for problem behaviors such as homelessness and substance abuse [17] and overreliance on the emergency room for health care [21]. Emerging adults with asthma are less likely to have a usual source of health care and more likely to visit the emergency department (ED) than older adolescents with asthma [22]. The transition to adult care can be abrupt and can occur with little preparation because age, rather than developmental maturity, triggers the transition. Given these barriers, it is not surprising that illness management deteriorates during emerging adulthood [18,23,24]. The research literature on interventions to improve asthma management is nearly nonexistent in emerging adults [25-27]. Results of studies with minority adults suggest that programs that target even just 1 or 2 aspects of asthma management (eg, adherence to medications) have largely been successful [28]. Thus, targeted interventions may have promise for improving morbidity and mortality rates in minority youth at highest risk for poor adherence and health outcomes. However, successful interventions must consider the target group’s distinctive developmental needs and unique culture [29]. This project tests a developmentally and culturally appropriate intervention for urban African American emerging adults (aged 18-29 years) with persistent asthma and poor adherence.

“eHealth,” “mHealth,” “telemedicine,” and “telehealth” have become buzzwords for the use of electronic information and mobile communication technology in health care and behavioral health [30,31]. Technology-based interventions cannot replicate the important human elements of traditional interventions. However, technology offers tremendous advantages in terms of reach, cost, anonymity, and time. We have developed and pilot tested a multicomponent, technology-based intervention (MCTI) specifically targeting adherence to controller medication in African American emerging adults that integrates 2 powerful technologies for behavior change—interactive computer-delivered intervention sessions and text messaging reminders between sessions. Each of these components is also tailored via text messaging based on an assessment of each participant’s experiences living with asthma. This method of data collection is called ecological momentary assessment or “EMA” [32]. EMA refers to a set of methods that allow a research participant to report on symptoms, affect, behavior, and cognitions in “real time” rather than rely on more traditional retrospective self-reporting such as with a questionnaire or interview [32]. Despite the rapid growth of technology in behavioral health, we know of no other published technology-based interventions that have specifically targeted African American emerging adults with asthma or any other chronic illness. We also are not aware of any other programs that have integrated multiple technologies for assessment, intervention, and intervention tailoring, which represent a major limitation, given that technology-delivered interventions for adults living with asthma generally have favorable results for asthma management and knowledge [33-36]. Technology-based interventions may be particularly well-suited for youth, including emerging adults, because they tend to use computers, cell phones, and text messaging so frequently in their daily lives [37-41]. Emerging adults, including urban minority youth, also tend to use multiple sources of technology.[42].

Motivational interviewing (MI) is a collaborative, goal-oriented style of communication with particular attention to the language of change. It is designed to strengthen personal motivation for and commitment to a specific goal by eliciting and exploring the person’s own reasons for change within an atmosphere of acceptance and compassion [43]. The theory underlying MI is consistent with evidence from other psychotherapy outcome research, which indicates that factors such as empathy, optimism, and congruence are strongly linked to client outcome [44]. Originally developed for use with substance abusers, MI adaptations for youth with other chronic conditions have shown strong effects for this brief intervention approach [45-49], and 1 meta-analysis suggested stronger effects in studies targeting minority populations [50]. MI is consistent with several models of health behavior change, including the information-motivation-behavioral (IMB) skills model that serves as the foundation of the current intervention [51]. Importantly, 1 common shortcoming of Internet-based behavior change interventions for asthma is lack of foundation in a theoretical model [52]. According to the IMB model, behavior change results from the joint function of 3 critical components: accurate information about risk behaviors (eg, risks of not taking asthma medications as prescribed) or their replacement health behaviors (eg, benefits of taking asthma medications), the motivation to change behavior, and the perceived behavioral skills necessary to perform the behavior (eg, self-efficacy). Thus, the intervention is intended to improve medication adherence behavior by targeting motivation and providing information about asthma management in an MI-consistent way. Furthermore, text messaging reminders promote the translation of information and motivation into behavior skills as they prompt the youth in real time about their plans for changing asthma management behavior. Overall, MI has been shown to hold great promise in promoting behavior and attitude change in people with health difficulties. However, one of the key issues when considering using MI in health settings is the amount of training that might be required for health professionals to use MI and whether implementing MI can fit within the demands of busy health settings [53]. The MCTI uses computer-delivered MI, which may offer some practical and logistical advantages over traditional person-to-person therapeutic delivery in busy health settings.

The purpose of this proposal was to test a brief MCTI, rigorously developed through a rigorous, multistep process with pilot funding from National Heart, Lung, and Blood Institute (NHLBI) to improve asthma controller medication adherence in urban African American emerging adults [54-57]. The MCTI is based on the IMB skills model, MI strategies, and EMA. The proposed project includes significant innovations that go beyond the current state of eHealth interventions by (1) using multiple technologies to extend the reach of the intervention and (2) individualizing the intervention for each participant through real-time assessment of his or her experiences living with asthma through EMA. All elements of the proposed study protocol were piloted in NHLBI R34 (1R34HL107664 MacDonell). Results suggested feasibility and acceptability of the study protocol as well as proof of concept in a pilot randomized clinical trial [55]. We are now ready to test the intervention in a randomized clinical trial. The proposed study will include 192 African American emerging adults with moderate to severe persistent asthma and low controller medication adherence recruited from clinic and ED settings. Half of the sample will be randomized to receive an MCTI targeting adherence to controller medication. The MCTI consists of 2 components: (1) 2 sessions of computer-delivered MI targeting medication adherence and (2) individualized text messaging focused on medication adherence between the sessions. At study start, participants receive 7 days of “real-time” data collection via text messaging (EMA) and daily diary. Data are collected on daily controller medication adherence, asthma symptoms, and barriers to asthma management and are used to tailor the intervention for each participant. The remaining half of participants (control group) will complete a series of computer-delivered asthma education modules matched for length and method of delivery of the intervention session. Control participants will also receive text messages between intervention sessions. Message content will be the same for all control participants and contain general facts about asthma. Youth will be recruited from the Detroit Medical Center, the only university-affiliated medical center in Detroit, Michigan. Detroit is an appropriate setting for this work as it has the highest percentage of African Americans of any major city in the United States. A 2013 report of the Michigan Department of Community Health found that asthma prevalence and asthma-related deaths in Michigan are above the national average and occur disproportionately in Wayne County where Detroit is located.

### Study Aims

#### Primary Aim

The primary aim of this study was to test the efficacy of a 2-session MCTI to improve controller medication adherence (primary outcome) and asthma control (secondary outcome) in African American emerging adults (aged 18-29 years) with poorly controlled asthma and low medication adherence.

#### Secondary Aim

The secondary aim of this study was to identify the mechanisms (mediators) of MCTI treatment effects on primary outcomes. Proposed mediators through which MCTI will exert its effects are derived from the IMB skills model: asthma treatment knowledge, self-efficacy, and motivation for adherence.

## Methods

### Study Overview

Participants will be randomized (1:1) to an MCTI targeting asthma medication adherence or to a comparison control condition. A repeated measures design (baseline and 1-, 3-, 6-, 9-, and 12-months postbaseline) will be used with the primary outcome being adherence to daily controller medications and the secondary outcome as asthma control. We expand on the pilot study [55] by testing the long-term effects over 1 year, which allows us to account for seasonal variability of symptoms in a young, urban population [58,59], to gain understanding of the long-term impact on adherence behavior, and to allow trajectory analyses of adherence.

### Overview of Multicomponent Technology-Based Intervention

The MCTI was tested in a pilot randomized controlled trial [54,60] and is an integration of several technology-based components. The intervention group will receive 2 sessions of computer-delivered MI via CIAS software (Interva, Detroit, MI) programmed to target adherence to medications. The intervention group will also receive text messaging adherence reminders between sessions. Both the computer-delivered sessions and text messages will be tailored to the participant using EMA.

Sessions are provided by an animated character (avatar, eg, “Peedy” the parrot). The participant picks their preferred character from 10 possible choices at the start of the session. All avatars were developed via focus groups with African Americans. Significant efforts have been made to ensure that the animated character delivers the intervention in a way that has high fidelity with the most recent edition of Motivational Interviewing (MI-3) [43]. MI-3 is specified by 4 processes: (1) engaging, (2) focusing, (3) evoking, and (4) planning. The intervention engages the youth with the avatar’s communication of empathy, optimism, and autonomy support. The intervention focuses the youth on adherence and relevant health behaviors with feedback on adherence, asthma symptoms, and tailored education. The avatar evokes both importance and confidence (key components of readiness or motivation) with MI strategies such as identifying pros of behavior change, affirmations to reinforce change talk and boost confidence, and identification of strengths and resources. The intervention also manages counter-change talk by having the avatar reflect without judgment and provide statements to emphasize autonomy. Finally, participants are guided in the planning process through goal-setting activities. Importantly, as with a face-to-face interaction with a human counselor, the interactions between the computer-delivered motivational intervention and the participant are synchronous and not reliant on feedback at the completion of the session. Small amounts of appropriate psychoeducation about potential improvements in asthma health outcomes that can result from improvements in asthma regimen adherence are integrated with the more purely motivational elements of the intervention. The provision of such information is consistent with the IMB model, which suggests that motivational approaches are most effective in the context of sensitively provided information about a health-related behavior [51], such as adherence. The length of the intervention sessions is about 30 min each, with the total duration of the visit (assessment and intervention) lasting about 1.5 hours.

Participants receive daily text messaging between the intervention sessions. The message timing and content are individualized based on EMA as well as participant response to the intervention. Those participants who indicate that they are “ready” to set the goal to take their medications as prescribed will receive text messages to remind them to take their medications. Those who indicate that they are less than ready (ranging from “not at all ready” to “somewhat ready”) to try and take their medications as prescribed can choose from a range of alternatives, including taking medications at least 50% of the time, to just think about taking medications, or to opt out of text messages and considering medications entirely. Text messaging content and timing for those who are not ready to take their medications are individualized based on the participant’s choice, ie, those who do not receive adherence reminders receive a daily message encouraging them to work toward their chosen goal.

The MCTI is tailored for each participant in several ways: (1) the computer-delivered intervention is an interactive program that is individualized based on MI principles, (2) participants in the intervention condition receive personalized feedback during the computer-delivered intervention sessions based on their recent asthma symptoms and medication use, and (3) text messages received between intervention sessions are tailored based on the medication routines and readiness to take medications as described by the participant.

### Control Condition

Effective comparison conditions must (1) include aspects or processes of treatment shared in common by behavioral interventions, (2) avoid processes specific to the experimental intervention, (3) avoid processes specific to other existing interventions, and (4) be credible enough to generate participant interest [61]. Control participants complete CIAS-delivered (Interva, Detroit, MI) asthma education modules matched for length, location, and method of delivery of the intervention session. These modules were created using content from the Asthma and Allergy Foundation of America and focus on facts and myths about asthma, controlling environmental factors such as asthma triggers, and pharmacologic management. Module content is consistent with the National Asthma Education and Prevention Program *2007 Expert Panel Report 3: Guidelines for the Diagnosis and Management of Asthma*. Control participants complete each module at their own pace and then complete a short quiz to assess their knowledge. Control participants also receive text messages between intervention sessions. Message content is the same for all control participants and contains general facts about asthma (not tailored). Messages timing is not tailored and is sent at the same time every day (4:00 PM—time chosen to avoid AM and PM medication times but not to interfere with sleep and school activities). This comparison condition controls for improvement because of nonspecific intervention factors such as positive expectancies due to entering treatment, positive regard and attention from being part of the intervention project, and having opportunities to learn about asthma and asthma treatment and matching for dose (EMA assessment, 2 sessions of software interaction, and daily text messages).

### Participants and Recruitment

The study will include 192 African Americans (aged 18-29 years) with moderate to severe persistent asthma requiring daily controller medications. We define persistent asthma according to 2007 NHLBI guidelines: level of symptoms, as defined by any of the following in the last 4 weeks: (1) use of any asthma medication more than 2 times a week; (2) daytime asthma symptoms such as wheezing, tightness of chest, coughing more than 2 times week, or waking up at night because of asthma more than 2 times a month. To be eligible, patients must report poor adherence to daily controller medications during eligibility screening. Poor adherence is defined as not taking medications “as prescribed” less than 80% of the time. This definition of underuse is consistent with previous work focused on underuse of asthma controller medications in children and adults. Participants must also live in the Detroit area (within 30 miles) and be able to complete questionnaires in English. Participants must also own or have access to a cellular phone for the duration of the study. We had no potential participants excluded because they lacked a cell phone with text messaging; moreover, nearly all had cell phone plans with unlimited texting. Thus, we expect few exclusions related to cell phone ownership. No exclusions will be made because of comorbid mental health problems (ie, attention-deficit/hyperactivity disorder depression), except thought disorder (ie, schizophrenia, autism), suicidality, or mental retardation. It is assumed that severe psychosis or suicidality may require management beyond the scope of our interventions. Youth with other chronic health conditions requiring ongoing medical intervention (eg, HIV, type II diabetes) will be excluded. Youth who have previously participated in our feasibility trials or pilot will also be excluded.

The proposed project will utilize the resources of the Department of Family Medicine, Clinical Research Service Center (CRSC), and Integrative Biosciences Center at Wayne State for recruitment, implementation, and retention. These centers have well-established recruitment and consenting practices; moreover, much effort went into streamlining our recruitment and consenting procedures during the R34 pilot. Health Insurance Portability and Accountability Act privacy practices require that contact regarding the study be made first by medical staff. We piloted recruitment procedures at multiple primary care and emergency care sites and have selected two for the randomized controlled trial: Detroit Medical Center Internal Medicine and emergency departments (EDs; Detroit Receiving Hospital and Sinai Grace). These sites were those where we actually found emerging adults in care for asthma or nonasthma-related issues. For recruitment in the ED, Clinical Research Service Center staff will recruit, preliminary screen, and obtain an authorization to contact form. Patients may be prescribed a controller medication and/or refill of medication(s) upon discharge of the ED, as is standard practice. In the internal medicine/ambulatory clinics, medical staff will screen and obtain an authorization to contact form. To expand our reach and enrollment, we are adding an additional ED site (Harper at Detroit Medical Center), several more federally-qualified health centers, and community/online recruitment approaches. Regardless of recruitment source, participants will be screened by a research assistant and consent using an in-person, written informed consent prior to enrollment and study entry.

### Procedures

All data collection and sessions will take place at clinic research space at Wayne State University iBio or in a private place (eg, the participant’s home). Data collection is conducted on the laptop computer with each questionnaire item read to the participant by the computer (headphones are provided for privacy). However, a human data collector is also present during the session to explain and demonstrate computer usage and troubleshoot any difficulties. Data collection occurs at baseline and at 1, 3, 6, 9, and 12 months, with intervention sessions at 1 week postbaseline and concurrent with the 1-month data collection. The 9-month assessment is limited and over the phone to reduce burden. Data collection includes measurement of pulmonary functioning, EMA, and computer-delivered questionnaires (CIAS). EMA is collected via daily text messaging and diaries before the computer-delivered sessions at baseline and at 3, 6, and 12 months. The Doser is attached to the primary MDI at baseline and at 6 and 12 months at the start of EMA and removed after each EMA period. We do not include EMA or Doser at every session to minimize participant burden (9-month follow-up is only CIAS (Interva, Detroit, MI), and data collection via phone call and EMA/Doser are staggered).

All sessions (including combined assessment or intervention session) will take less than 1.5 hours to complete (we reduced number of questionnaires based on participant feedback). Participants will be randomized following the baseline session. For the intervention group, the intervention authoring ability is built on top, so responses to assessment questions feed directly into the intervention to begin the intervention tailoring. Control group participants receive the same questionnaires, EMA, and pulmonary functioning assessment for data collection followed by computer-delivered asthma education matched for length of time of the intervention. Because the assessment is computer administered, there is no data collection staff per se for written measures. Research staff responsible for recruitment and retention will be blind to treatment status to the extent possible in a behavioral trial. The software will randomly assign participants to conditions in a 1:1 ratio and notifies the participants of their randomization status. To minimize attrition, we will use approaches that have been successful in our studies in retaining urban, minority samples such as keeping in regular contact with participants via letters, calls, and texts, requesting alternate contacts in case phone numbers change, and so forth.

Measures are collected at baseline and at 1, 3, 6, 9, and 12 months (see Table 1). Psychological distress and demographics are collected only at baseline. Pulmonary function testing (PFT) and Doser are collected at baseline and at 6 and 12 months. EMA is not obtained at 1 month because of study design or at 9 months to reduce participant burden. All measures have been found to be reliable and valid.

**Table 1:** Primary and secondary outcome measures. EMA: ecological momentary assessment; IMB: information-motivation-behavioral.

Measure		Description of measure
**Primary outcome: medication adherence**		
	Medication adherence (EMA)	Text messages daily to prompt for adherence to controller medication. Participants will also complete a diary at the end of each day to assess asthma symptoms and barriers.
	Medication adherence [62] (written measure)	12-item, yes/no response measure asks about controller medication use in the last 3 months (Have you forgotten to take medications as prescribed, etc.).
	Medication adherence (objective measure) [63]	A Doser tracking device will be used (with covert display mode) to measure number of doses during the EMA period at baseline and at 6 and 12 months [64]. Prompts have been added to check that devices are attached and functional.
**Secondary outcome: asthma control**		
	Asthma symptoms (EMA)	Text messages daily to prompt for asthma symptoms and a text each morning to ask for total symptoms experienced the previous day and night. Participants also complete a diary at the end of each day to assess mood and impact of asthma on activity level and daily life.
	Asthma Control Test (ACT) [65]	A 5-item questionnaire. Scores range from 5 (poor control of asthma) to 25 (complete control of asthma), with higher scores indicating greater asthma control. An ACT score >19 indicates well-controlled asthma.
	Pulmonary functioning	Forced expiratory maneuvers (forced expiratory volume in 1 second and forced viral capacity) will be obtained with a calibrated recording spirometer (KoKo portable device) at baseline, and at 6 and 12 months.
**Proposed mediators**		
	Asthma Knowledge, Attitudes, and Self-Efficacy Questionnaire (KASE-AQ) [66]	60 items, using a Likert scale response set, assess knowledge regarding asthma, their attitudes about their asthma, and their self-efficacy regarding their perceived ability to control their asthma consistent with the IMB skills model.
	Motivation for adherence	Internal and external motivation for asthma medication adherence will be measured consistent with the IMB skills model.
**Baseline descriptive measures**		
	Alcohol, Smoking, and Substance Involvement Screening Test (ASSIST) [67]	The ASSIST was developed for the World Health Organization to detect psychoactive substance use and related problems in primary care patients. Only tobacco and marijuana use will be assessed.
	Brief Symptom Inventory (BSI) [68]	The BSI-18 measures psychological distress (anxiety, depression, and somatic complaints).
**Participant satisfaction**		
	Client satisfaction	Participants will complete a brief satisfaction questionnaire at the end of session 2.

### Statistical Analyses

The intervention effect will be assessed in 3 steps. First, assess comparability of the intervention and control condition. Despite random sampling, participants in the actual sample may not always be comparable. Baseline differences in mean scores of medication adherence and asthma control and demographic characteristics (eg, age, gender, and race) and attrition to follow-up between the intervention and control groups will be examined using chi-square test (for categorical variables) and analysis of variance (for continuous variables). Variables that significantly differ between intervention and control groups will be used as covariates in multivariate analysis. Second, bivariate analyses will be used to assess potential differences between intervention and control groups on outcome measures (medication adherence and asthma control measured as a count variable) at baseline and each follow-up. To examine the effect of the intervention on these outcomes, we compared the difference in medication adherence and asthma control between the intervention and control groups using Wilcoxon rank-sum test (instead of *t* test), as these outcomes are usually non-normally distributed. Asthma control will be further categorized into “well-controlled” versus “poorly controlled” asthma. The difference in the proportion of “well-controlled” asthma between the intervention and control groups will be assessed using chi-square test. The difference in proportion of “well-controlled” asthma between baseline and follow-up visits within each study group will be examined using McNemar test. Finally, significant findings will be verified using a multivariate approach. The intervention effect on medication adherence and asthma control will be further assessed using mixed-effects modeling, controlling for potential confounding factors (eg, age, race, gender, and intervention sites), possible baseline differences in outcome measures, and the clustering effects of repeated measurements. To use the mixed-effect modeling analysis in assessing intervention effect, an interaction term (intervention group by time) will be included together with other covariates. A significant beta coefficient of the interaction term at *P*<.05 level will be used as evidence supporting the intervention effect.

Specifically, mixed-effects models using PROC MIXED SAS procedure will be conducted to examine the intervention effect on medication adherence and asthma control. For asthma control outcome (“well-controlled” vs “poorly controlled” asthma), we will also conduct generalized mixed models (PROC GLIMMIX). Missing data (missing at random) are handled using full information maximum likelihood estimation in these mixed-effect models. If data are not missing at random, we will conduct a sensitivity analysis, which allows us to investigate possible violations of the missing data at random assumption. When the amount of missing data is large (25%), which is very unlikely to occur based on our data collection experience in the previous R34 grant, we may consider the last observation carried forward method in the longitudinal data analysis. All the analyses will be conducted using SAS 9.4 statistical software package.

Structural equation modeling (SEM) will be used to examine the extent to which the targeted variables (eg, IMB skills) mediate the intervention effect. The purpose is to provide further evidence supporting the theory-based intervention. The analysis plan expands on the procedures outlined by Baron and Kenny [69], making use of causal modeling. Simple correlation and path models will be used first to explore variables that may mediate the intervention on outcome measures. SEM models will thus be constructed based on results from the simple analysis. To build a parsimonious SEM mediation model, empirical Bayes estimates of intra-individual change will be estimated for both mediating constructs and outcome variables. SEM analysis will be performed with the software M-Plus. The model will be evaluated using several model fit indices, including standardized root mean square residual (SRMR), comparative fit index, and root mean square error of approximation (RMSEA). Values for the SRMR range from 0 to 1 with well-fitting models obtaining values less than .05 [70,71]. For comparative fit index, a cutoff criterion of ≥.95 will be used as indicative of good fit [72]. For RMSEA,.01, .05, and .08 will be used to indicate excellent, good, and mediocre fit, respectively [73].

It is critical to understand who responds to a brief intervention and who might require more intensive treatment. We will use developmental trajectory analysis to explore subgroups of participants with similar adherence and control trajectories over 12 months. This approach is preferable to a priori specifying the type or number of trajectories or relying on ad hoc categorization and has been recently applied to understanding clinical trials outcomes with children and adolescents. Some studies use only 2 trajectories of response and nonresponse and some find more (eg, improvers, slow improvers, relapsers, and responders). We will then use logistic regression to assess demographic and hypothesized predictors of treatment response (substance use, mental health, barriers, treatment site, and engagement in health care). Binary logistic regression may be used for 2 trajectories or to compare the most improved or least improved with others, whereas multinomial logistic regression may be used to compare multiple trajectories.

### Potential Problems and Alternative Strategies

Differential loss to follow-up is a threat to internal validity to assess the effect of an intervention program. Possible barriers to project completion are high rates of no-shows at clinic visits or refusals to participate during ED recruitment, which would interfere with study recruitment and retention. Recruitment procedures and strategies were a primary focus of the R34-funded pilot, as we recognize that this population can be difficult to find in care and to recruit. In addition, we have strong collaborations in place through the Clinical Research Service Center and nurse recruiter to ensure recruitment success. Another possible issue is retaining youth in the study for the extended period (12 months). Multiple techniques are used to increase the likelihood that participants will keep their data collection appointments, including advanced scheduling, multiple reminder letters, and phone reminders. We piloted successful procedures and strategies for recruitment and retention during our pilot work, and our research center has a strong history of successful work with at-risk urban populations. We were also able to retain youth from 1- to 3-month follow-ups (with no activity between these data collections) and to have them initiate 3-month EMA without a face-to-face data collection. For the proposed trial, we will be seeing participants face-to-face to attach the Doser device at the start of the EMA period, which we anticipate will help ensure continued participation. Participants who do not complete intervention or control sessions will still be asked to provide follow-up data. When follow-up data are not available, the mixed model approach will use maximum likelihood estimates and all available data so no participants would be excluded. Thus, the intent-to-treat analyses will include all randomized participants, regardless of the intervention dose delivered. If missing data are not related to the outcome measures (missing at random), we will impute the missing data using advanced methodology [74]. Threats to internal validity of the study also may arise without sufficient attention to quality assurance of data collection and intervention delivery. Possible technological difficulties with the software, text messaging, or the server is another concern. Although these problems were minimal and remediated in the pilot study, we now have specific protocols and budget for technical support.

## Results

The research protocol R01HL133506 (MacDonell) was funded by the NHLBI for the period September 1, 2016, through August 31, 2021. Notice of award was received on November 4, 2016. Enrollment began in June 2017 and will continue through February 2020. As of April 2018, 41 participants have been consented and enrolled. The final proposed sample is N=192.

## Discussion

Youth randomized to the MCTI will show significantly greater improvements from baseline to postintervention in adherence as assessed by (1) self-reported doses missed, daily medication adherence (EMA), and monitoring using an adherence tracking device and (2) asthma control as assessed by symptom frequency from EMA and lung functioning (forced expiratory volume in 1 second) than youth randomized to the control condition delivered by the same platform and matched for dose.

Older adolescents and young adults have largely been ignored in the asthma intervention literature, although they are at highest risk for poor illness management and health outcomes. This project will test a brief, technology-based intervention specifically targeting adherence to asthma controller medications in an under-researched population, urban African American emerging adults. If successful, our MCTI aimed at improving adherence to asthma medications has the potential to improve quality of life of minority emerging adults with asthma at relatively low cost. Furthermore, it could be eventually be integrated into clinical settings and practice to reach a large number of emerging adults with asthma.

## Abbreviations

ACT: Asthma Control Test
ASSIST: Alcohol, Smoking, and Substance Involvement Screening Test
BSI: Brief Symptom Inventory
ED: emergency department
EMA: ecological momentary assessment
IMB: information-motivation-behavioral
MCTI: multicomponent technology-based intervention
MI: motivational interviewing
RMSEA: root mean square error of approximation
SEM: structural equation modeling
SRMR: standardized root mean square residual
